# Supplementary material for: Iterative improvement in the automatic modular design of robot swarms
Source: PeerJ Comput Sci. 2020 Dec 7;6:e322. doi: 10.7717/peerj-cs.322 (PMC7924708; doi:10.7717/peerj-cs.322)
Supplement: Supplemental Information 3 [file peerj-cs-06-322-s003.zip › argos3/doc/api/standalone/a00473_source.html]

ARGoS: plugins/robots/foot-bot/simulator/footbot\_proximity\_default\_sensor.h Source File


- Main Page
- Related Pages
- Namespaces
- Classes
- Files

- File List
- File Members

# plugins/robots/foot-bot/simulator/footbot\_proximity\_default\_sensor.h

Go to the documentation of this file.

```
00001 
00007 #ifndef FOOTBOT_PROXIMITY_DEFAULT_SENSOR_H
00008 #define FOOTBOT_PROXIMITY_DEFAULT_SENSOR_H
00009 
00010 #include <string>
00011 #include <map>
00012 
00013 namespace argos {
00014    class CFootBotProximityDefaultSensor;
00015 }
00016 
00017 #include <argos3/plugins/robots/foot-bot/control_interface/ci_footbot_proximity_sensor.h>
00018 #include <argos3/plugins/robots/generic/simulator/proximity_default_sensor.h>
00019 
00020 namespace argos {
00021 
00022    class CFootBotProximityDefaultSensor : public CCI_FootBotProximitySensor,
00023                                           public CSimulatedSensor {
00024 
00025    public:
00026 
00027       CFootBotProximityDefaultSensor();
00028 
00029       virtual ~CFootBotProximityDefaultSensor();
00030 
00031       virtual void SetRobot(CComposableEntity& c_entity);
00032 
00033       virtual void Init(TConfigurationNode& t_tree);
00034 
00035       virtual void Update();
00036 
00037       virtual void Reset();
00038 
00039    private:
00040 
00041       CProximityDefaultSensor* m_pcProximityImpl;
00042 
00043    };
00044 
00045 }
00046 
00047 #endif
```

---

Generated on 10 Jul 2018 for ARGoS by 
 1.6.1
